# Supplementary material for: All-at-once RNA folding with 3D motif prediction framed by evolutionary information
Source: Nat Methods. 2025 Oct 3;22(10):2094–106. doi: 10.1038/s41592-025-02833-w (PMC12510878; doi:10.1038/s41592-025-02833-w)
Supplement: Supplementary file 3 — Source code to run CaCoFold-R3D. Data and results. [file 41592_2025_2833_MOESM3_ESM.gz › supplemental_material/results/CaCoFold-R3D/CaCoFold-R3D_Rfam_vshuffle/RF02201_TTC28-AS1_4.R2R.sto.pdf]

RF02201_TTC28-AS1_4

5′ – Y G U G C ● Y R Y Y U R R Y C C R ● C U G ● C ● Y R C U G C Y ● Y G G G C R C Y ● G G C U G A G G G U G G C A G ● R R Y R C U ○ R G C U A A C C C A A A G U G A C A Y R U G C C U G G Y G G G A Y R Y U C A G ● C U ● Y R Y A R A
